# Culture Positivity of CVCs Used for TPN: Investigation of an Association with Catheter-Related Infection and Comparison of Causative Organisms between ICU and Non-ICU CVCs

**DOI:** 10.1155/2012/257959

**Published:** 2012-04-19

**Authors:** Criona Walshe, J. Bourke, M. Lynch, M. McGovern, L. Delaney, D. Phelan

**Affiliations:** ^1^Department of Intensive Care Medicine, Mater Misericordiae University Hospital, Eccles Street, Dublin 11, Ireland; ^2^Department of Microbiology, Mater Misericordiae University Hospital, Eccles Street, Dublin, Ireland; ^3^Department of Public Health Medicine, Geary Institute, University College Dublin, Belfield, Dublin 4, Ireland

## Abstract

A relationship between central venous catheter (CVC) tip colonisation and catheter-related blood-stream infection (CRBSI) has been suggested. We examined culture positivity of CVC tips (colonised and infected CVCs) in a total parenteral nutrition (TPN) population. Our aims were to define the relationship between culture positivity and CRBSI, and to compare causative organisms between culture positive and CRBSI CVCS, and between ward and ICU CVCs. All patients receiving TPN via non-tunnelled CVCs during the study (1997–2009) were included. All CVC tips were analysed. Data were collated contemporaneously. A TPN audit committee determined whether CVC tip culture positivity reflected colonisation/CRBSI using CDC criteria. 1,392 patients received TPN via 2,565 CVCs over 15,397 CVC days. 25.4% of CVCs tips were culture positive, of these 32% developed CRBSI. There was a nonsignificant trend of higher Gram negative Bacilli isolation in ICU CVCs (*P* = 0.1), ward CVCs were associated with higher rates of staphylococcal isolation (*P* = 0.01). A similar pattern of organisms were cultured from CRBSI and culture positive CVCs. The consistent relationship between CRBSI and culture positive CVCs, and similar pattern of causative organisms further supports an aetiological relationship between culture positive CVC tips and CRBSI, supporting the contention that CVC culture-positivity may be a useful surrogate marker for CRBSI rates.

## 1. Introduction

Septic complications of central venous catheters (CVCs) remain a significant cause of patient morbidity and mortality both in the intensive care unit (ICU) and on general hospital wards. Approximately 25% of CVCs inserted have been reported to become colonised [1], with rates of catheter-related blood stream infection (CRBSI) varying between 0% and 11% [2–8]. On the basis of varied items of data [1, 9, 10], there is preliminary evidence that colonisation of CVCs may play a role as a pathogenic mechanism for the development of CRBSI.

There is limited data on CVC colonisation and CRBSI rates outside the ICU, in part due to the perceived high usage of CVCs within the ICU setting. However, because of the larger population of patients managed at ward level, the majority of CVCs may in fact be inserted and maintained in patients located outside of the ICU on general wards, as high as 70% in one survey [11]. This suggests that hospital wide surveillance of CVC complications should be a priority and focus is now turning toward the measurement of CRBSI incidence and epidemiology in CVCs inserted in patients at ward level [12]. It has also been contended, but with limited evidential support [13–15], that CVCs inserted in ICU patients may have different incidence of infectious complications relative to CVCs inserted in ward patients, with different patterns of causative organisms. This has obvious implications for best CVC insertion practice and empiric antimicrobial therapy in the event of CVC-related sepsis.

The aims of this 12-year study were to document the proportion of CVCs in a hospital-wide TPN population that became tip culture positive (incorporating colonised and infected CVCs), and to examine the consistency of any relationship between culture positivity of CVC tips and development of CRBSI. We also aimed to define the microbiological pattern of causative organisms of culture positive and CRBSI CVCs and to compare the pattern of organisms from CVCs inserted in the ICU with CVCs inserted in the operating theatre (OT) for general hospital ward patients. 

## 2. Methods

The study was set in a 525-bed city centre tertiary referral institution. The TPN service is run from the ICU under the clinical direction of the intensive care consultant staff and receives referrals from all disciplines. 

All patients commenced on TPN are subject to a hospital audit process and a monitoring database was established in 1990. Data were entered prospectively into TPN audit records by the dedicated TPN surveillance nurse practitioner. All patients referred to the hospital TPN service for placement of a standard CVC for administration of TPN between January 1997 and January 2009 were included. Tunneled CVCs, peripherally inserted central venous catheters (PICC), peripheral intravenous cannulae, and CVCs that had been inserted in other hospitals were excluded, as were patients receiving home TPN. No antibiotic or otherwise impregnated CVCs were in use during the study period. 

A TPN audit committee, comprising the intensive care consultant and senior registrar, TPN surveillance nurse, and Microbiology consultant, met quarterly throughout the study period [16]. Data regarding all complications of TPN CVCs, including septic complications, were collected by the TPN surveillance nurse and these records were reviewed by the TPN audit committee. The committee assigned a diagnostic category (noninfected, colonised, or CRBSI) to all catheters potentially responsible for episodes of sepsis occurring in the hospital TPN population, using CDC compatible definitions [17]. 

All CVC tips from TPN patients were cultured, regardless of whether removal was because the CVC was no longer required or because of suspected CVC-related sepsis. Catheter tip culture positivity (CP) was defined as growth of >15 colony forming units (cfu) per mL from the inside of a distal catheter segment using semiquantitative microbiological techniques as described by Maki et al. [18]. 

Culture positive CVCs were deemed to be “*colonised*” when catheter tip was culture positive but not associated with clinical sepsis [17, 19]. “*CRBSI*” episodes were diagnosed in patients with culture-positive CVCs meeting the CDC criteria for CRBSI current at the time, outlined as follow [17].

- isolation of an organism by semiquantitative culture from a catheter segment in a patient with clinical sepsis [20], and
- isolation of the same organism from the patient's blood, or
- defervescence of sepsis following removal of the catheter.

Care and maintenance of CVCs were standardised in the hospital. The subclavian vein was the recommended anatomical approach provided the operator was comfortable with this in the individual patient, and there were no contraindications to this approach. The CVCs were placed by predetermined registrars in intensive care/anaesthesia under TPN service consultant supervision. All CVCs were inserted either in the ICU (intensive care and high dependency units) or in the OT for TPN administration in general ward patients (OT/ward CVCs). Patients received standardised care as outlined previously [21]. CVCs were carefully removed by nurses using written protocols involving initial antiseptic cleaning, the tip being removed under aseptic conditions and sent for culture in all situations. 

 Organisms isolated from CVC tips were categorised as follows:

1. methicillin-sensitive *Staphylococcus aureus* (MSSA)
2. coagulase-negative *Staphylococcus* (CNS)
3. methicillin-resistant *Staphylococcus aureus* (MRSA)
4. gram-negative Bacilli (GNB)
5. fungi
6. enterococci
7. polymicrobial causes.

### 2.1. Statistical Analysis

The data was collated from the specific TPN audit records and transferred into spreadsheets. All CVCs that became culture positive, incorporating colonised CVCs and those responsible for CRBSI, were examined. Statistical analysis was by means of paired *t* tests, comparing the incidence of each causative organism. 

Incidence rates for CRBSI and culture positivity were also compared using paired *t* tests. In order to examine whether there were any changes in infection rates over the course of the study period, ordinary least squares regressions were conducted. 

In each regression, the incidence rate in each group (CRBSI, culture positive) for a particular year was the dependent variable, with the sole independent variable being the year. The difference in rates between the two groups was also used as a dependent variable. These three regressions allow us to identify whether, for each outcome (viz. the infection rate in the CRBSI, the incidence rate in the culture-positive group, and the difference in incidence rates between CRBSI and culture-positive groups), whether there was any statistical reduction or increase over time. The whole analysis was carried out using Stata 10. 

## 3. Results

**Figure 1:**
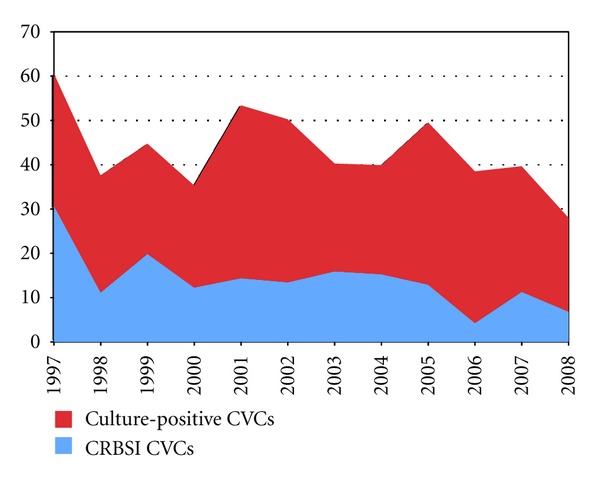
Annual incidence of culture-positive CVCs and CRBSI expressed as episodes per 1000 CVC days.

Throughout the twelve-year study period, spanning January 1997 to January 2009, a total of 1392 patients received TPN via 2,565 CVCs over 15,397 CVC days. 25.4% (651/2565) of CVCs inserted for TPN administration throughout the study period became culture positive. Of these culture positive CVCs, 68% (441/651) were colonised and 32% (210/651) were associated with the development of CRBSI.  Figure 1 depicts the annual incidence of culture-positive CVCs subdivided into colonised CVCs and those responsible for CRBSI expressed as episodes per 1000 CVC days. 

Table 1 shows that the proportion of culture positive CVCs that were associated with CRBSI averaged 32% (derived from mean annual number of episodes = 17.5/54.25), the difference between culture positive and CRBSI was statistically significant at −36.75 (SE 1.83), (*P* < 0.01). 

Table 2 shows the decline in the proportion of CVCs that became culture positive in the study period. In contrast to the previously reported significant fall in CRBSI rate (the regression analysis estimates a significant fall of 1.182 on average for each year in the study period, (*P* < 0.05) [21]); the decline in culture-positive CVCs (0.465) was not statistically significant (*P* > 0.05). 

Table 3 depicts the pattern of organisms isolated from CVCs that became culture positive and those responsible for development of CRBSI. It demonstrates that a similar pattern of organisms was isolated from CVC tips of both groups for all categories of causative organisms with the exception of enterococci, which were statistically more likely to be isolated in culture positive than CRBSI CVC tips (*P* = 0.04). 

Table 4 displays causative organisms isolated from cuture positive CVCs in ICU and OT/ward CVCs, and shows that the proportion of culture positive CVCs that grew staphylococci (incorporating MSSA, CNS and MRSA) was significantly higher in OT/ward CVCs (74.7%) compared to ICU (64%), (*P* = 0.01). More specifically, MSSA isolation was significantly higher in the OT/ward CVCs compared to ICU (*P* < 0.01). There were no other significant differences in the pattern of organism isolated between ICU and OT/ward CVCs.

## 4. Discussion

In this large study of hospital-wide TPN patients, we examine the prevalence of CVC tip culture positivity, incorporating both those CVCs that became culture positive in the absence of systemic sepsis (colonised CVCs) and culture-positive CVCs causative of systemic infection (CRBSI CVCs), in a hospital-wide TPN service. We do not recommend that routine culture of CVC tips be carried out, however, given that the policy and practice was to culture all CVC tips used for TPN administration throughout the study period in such a large, hospital-wide TPN population, we were afforded the unique opportunity to carry out the current analysis allowing us to fully define the relationship between culture positive CVC tips and subsequent development of CRBSI using standard CDC diagnostic criterion. Our results demonstrate that 25% of standard, nontunnelled CVCs inserted for TPN administration became culture positive throughout the study period, and that there was a consistent relationship between culture positivity and the development of CRBSI (Table 1). 

The terminology utilized in much of the literature to describe colonised and infected CVCs is inconsistent as many studies appear to use the word “colonised” to include all catheters that are tip positive (by local/standard criteria) [1, 2, 6, 22–24]. However, these studies then describe the proportion of these “colonised" CVCs that resulted in systemic infection (CRBSI) thus creating an inherent contradiction as CVCs responsible for CRBSI episodes are thus being included in the “colonised” group. We concur with the clarifying terminology utilized in a recent study examining CRBSI/colonisation in arterial cannulae which utilises the term “culture positive” to cover both categories (colonised CVCs and those CVCs that resulted in CRBSI) [25], as it is descriptively precise and we have used this terminology here. 

In a review by Marik [1], it is suggested that approximately 25% of CVCs becomes colonised. However, this estimate was based on evidence using varying terminology, from a variety of clinical settings including arterial catheters, CVCs, and antibiotic-impregnated CVC studies [25–27] and there was considerable variability in colonisation and infection rates. The current study, of a large population of uniform, nontunneled, standard, nonantibiotic impregnated CVCs, confirms a *culture positive*, not colonization, rate of 25.4% for all CVCs.

There is limited published evidence to support the relationship between culture-positive CVCs and the subsequent development of CRBSI. In the review mentioned above, Marik has suggested that between 20–30% of “colonised” catheters results in CRBSI [1]. In a large meta analysis Rijnders et al. [28], the authors demonstrate a linear correlation between catheter tip “colonisation” and CRBSI. This correlates with the evidence of the current study where 32% of TPN CVCs that became culture positive resulted in the development of CRBSI, a relationship that was consistent throughout the study period (Table 1). This study provides further circumstantial evidential support for CVC tip colonisation as an important pathogenic mechanism for CRBSI in that it demonstrates a similar pattern of organism growth from both colonised and CRBSI CVCs (Table 3). 

The decline in CVC culture positive rates seen (Table 2, an estimated reduction of 0.465 on average per year during the study period, *P* > 0.05) did not reach statistical significance in this study but is likely related to the known significant decline in CRBSI rate in this population (Table 2, an estimated reduction of 1.182 on average per year during the study period, *P* < 0.05) and reflective of the value of a surveillance nurse implementing hygiene and other preventative measures as a probable means of reducing CRBSI [21]. This decline in CRBSI correlates with data presented in the literature describing falling CRBSI rates, resulting in overall CRBSI incidences tending to be generally low, in the region of 5 episodes per 1000 CVC days [29]. 

With such a low prevalence of CRBSI, it is difficult to detect differences in outcome measures such that extremely large numbers of patients and CVCs would need to be recruited in future studies to demonstrate significant differences between groups, and others have alluded to this difficulty [28]. This has led to the suggestion of use of CVC tip colonisation as a representation, or surrogate marker, for CRBSI in many clinical studies [30–34]. This use of colonisation data for CVC tips has been demonstrated to be a practicable endpoint in future studies by Rijnders et al. in a large review of the current evidence [28]. Given that the current study provides further evidential support for the relationship between culture positivity and CRBSI and presuming the consistency of this relationship is confirmed in further studies, it supports the auditing of CVC tip culture positivity as a surrogate surveillance marker for CRBSI which may provide a more dynamic measurement of trends than CRBSI measurement alone—especially where numbers of CVCs inserted are low. However, this would entail culturing all removed CVCs, regardless of whether or not catheter-related infection was suspected, and the issue of cost versus benefit would require analysis in any hospital/laboratory considering such a regular process. 

It has been proposed that TPN, being a potential culture medium, is an independent risk factor for CRBSI [35–38]. However, there is a paucity of studies related to CVC colonisation and CRBSI and in patients receiving TPN via short-term CVCs. Of the limited prospective studies of CRBSI among TPN patients, levels reported are diverse with CRBSI rates ranging from 55.2% in one study [39] and 11.46% in another [37]. Insofar as one can judge the current evidence base, the proportion of culture-positive CVCs that resulted in CRBSI was little different from those quoted for CVCs for general use. Suggestions that TPN is an independent risk factor for CRBSI (and perhaps culture positivity) are therefore questioned by data from this study. 

In a CDC survey published in 2003, 55% of patients in the ICU had a CVC in situ, compared with 24% of ward patients [11]. However, because of the much larger population of patients located on general hospital wards, the majority of CVCs in use were in fact inserted in patients who were located outside of the ICU (70%) [11], and this is probably the case for most institutions. This has implications for the care and use of CVCs. One study reports higher risks of breaches in CVC care to occur on wards [40]. In another study, non-ICU patients tended to have CVCs in situ for longer periods of time [41]. Both of these are factors that place patients at higher risk of CRBSI and focus is now turning toward the measurement of CRBSI incidence and epidemiology in CVCs inserted in patients at ward level [12]. Despite this, there are very limited studies examining CVC colonisation and CRBSI among non-ICU populations, and less is still that compare these in a homogenous population. The current study is unique in that it provides comprehensive data on the microbiology of CVC tip colonisation and CRBSI in CVCs inserted in patients both in the ICU and at ward level. 

In a recent study by Zingg et al. [42], similar rates of CRBSI were determined among ICU and non-ICU patients, with most CRBSI being due to gram-positive organisms (60%), followed by gram-negative organisms (13%). However, they do not separate out causative organisms in ICU and non-ICU CVCs. In fact, we could find no studies comparing causative organisms for colonised or CRBSI CVCs in ICU and non-ICU patients within the same institution. Causative organisms for CRBSI among ICU patients tend to most commonly be due to gram-positive organisms between 55.4–88%, followed by gram-negative organisms 8.5–30.1% and candida 2.8–11.7% [10, 43–45]. Other studies examining CRBSI in non-ICU populations determined gram-positive organisms to be the most common causative organisms responsible for between 40–57% of CRBSI episodes [13, 46, 47]. Gram-negative organisms were causative for 17% of CRBSI in these studies of non-ICU patients followed by candida in 12–14% [13, 46]. 

These data correlate with the results of the current study, with gram-positive organisms being the most common causative organism in both culture-positive and CRBSI CVCs (Table 4) in both the ICU and at ward level, followed by gram-negative organisms with a trend (although nonsignificant) toward increased incidence among ICU CVCs. The use of vancomycin as empiric antibiotic therapy to treat suspected CRBSI is further supported by the current study. However, Table 4 shows that this consideration appears to be even more appropriate in OT/ward CVCs where there was a significantly higher (*P* < 0.01) staphylococcal isolation rate (74% of CVCs) than in ICU (64%) and the rate of MSSA isolation specifically was also significantly higher (*P* < 0.01) in OT/ward patients (4.6%) than in ICU (0.3%). This may reflect the slightly (but not significantly) higher GNB, enterococcal, and polymicrobial isolates from ICU CVCs. 

Strengths of this study include its large size, the systematic tracking of prospectively collected data on 1392 patients over 15,397 CVC days in patients receiving TPN, and the use of the same diagnostic microbiological criteria (for catheter tip positivity) and the same multidisciplinary process using CDC criteria for CRBSI diagnosis over the 13 years of the study. Weaknesses are the observational nature of the study design, the questionable relevance of CVC colonisation as a clinical entity, and the possibility of CVC culture positivity being merely representative of contamination at the time of CVC removal. However, the standard procedure for CVC removal outlined in the methods and the TPN surveillance nurse may have provided protection against this. Furthermore, although the CVC tip culture methodology used has been arguably superseded, its 13 years of consistent use lent validity to this study. 

In conclusion, this large study of TPN CVCs demonstrates a consistent relationship between CVC culture positivity and CRBSI, and that the organism pattern for culture-positivity and CRBSI is similar thus supporting the contention that colonisation is related to CRBSI and that CVC culture positivity may be a useful surrogate marker for CRBSI rates [26]. Staphylococci are the primary organism causing culture positivity in all CVCs and are significantly more likely to be cultured in OT/ward patients than ICU patients. This study also highlights, that as 25% of CVCs became culture positive and 32% of these were responsible for development of CRBSI, continued vigilance to reduce infection through prevention methods remains important.

## Figures and Tables

**Table 1:** Comparison of CRBSI versus culture positive CVCs (episodes per year).

Variable	Mean annual number of episodes	Standard error
CVCs that developed CRBSI	17.5	1.98

CVCs that became culture positive	54.25	2.15

Difference	−36.75	1.83

*P* < 0.01. Legend: The difference between CP and CRBSI episodes of 36.75 (SE 1.83) over the 12 years of observation is statistically significant, suggesting a consistent pattern of culture positive CVCs that resulted in development of CRBSI.

**Table 2:** Regression analysis using ordinary least squares (OLS) expressing the decline in culture-positive and CRBSI rates per year over 12 years.

	Year	Standard error
Culture positive	−0.465	(−0.64)
CRBSI	−1.182*	(−0.47)

*P < 0.05.

**Table 3:** Pattern of organisms isolated in culture-positive and CRBSI CVCs.

Causative organisms	Culture-positive CVCs Mean % annual incidence ± Std Dev	CRBSI CVCs Mean % annual incidence ± Std Dev	*P *value
All staphylococci (CNS, MSSA, MRSA)	69.2 ± 8.0	69.7 ± 16.22	0.9
CNS	59.2 ± 4.5	57.5 ± 11.4	0.6
MSSA	1.7 ± 1.3	1.0 ± 1.9	0.3
MRSA	8.3 ± 6.8	11.1 ± 11.6	0.5
Gram-negative Bacilli	11. 8 ± 6.3	12.2 ± 8.9	0.9
Fungi	5.7 ± 2.8	6.1 ± 4	0.8
Enterococci	2.4 ± 1.7	0.8 ± 1.9	0.04
Polymicrobial	10.8 ± 4.9	11.2 ± 8.5	0.9

**Table 4:** Culture-positive CVCs by category of isolated organism comparison of critical care and ward areas.

Causative organisms	ICU Mean % annual incidence ± Std Dev	OT/ward areas Mean % annual incidence ± Std Dev	*P* value
All staphylococci (CNS, MSSA, MRSA)	64 ± 10.3	74.7 ± 7.2	0.01
CNS	55.9 ± 7.9	62.4 ± 8.4	0.10
MSSA	0.3 ± 1	4.6 ± 2.1	<0.01
MRSA	7.9 ± 8.8	8.6 ± 6.7	0.80
Gram-negative Bacilli	14.6 ± 9.3	9 ± 4.8	0.10
Fungi	5.9 ± 3.8	5.6 ± 4.4	0.80
Enterococci	3.4 ± 3.1	1.5 ± 1.9	0.10
Polymicrobial	12.1 ± 4.6	9.2 ± 5.7	0.20
